# Prevalence and concordance of HER2-low and HER2-ultralow status between historical and rescored results in a multicentre study of breast cancer patients in China

**DOI:** 10.1186/s13058-025-02001-0

**Published:** 2025-03-25

**Authors:** Hong Lv, Junqiu Yue, Qingfu Zhang, Fangping Xu, Peng Gao, Haifeng Yang, Xiu Nie, Lingfei Kong, Guanjun Zhang, Jianming Li, Shiwei Xiao, Hongmei Wu, Aiyan Xing, Min Hong, Jun Fan, Huijuan Guan, Peilong Cao, Hengli Ni, Wentao Yang

**Affiliations:** 1 Department of Pathology, Fudan University Shanghai Cancer Center, 270 Dongan Rd, 270, Xuhui District, Shanghai, 200032 China; 2 Department of Pathology, Hubei Cancer Hospital, Wuhan, Hubei China; 3 Department of Pathology, The First Hospital of China Medical University, Shenyang, China; 4 Department of Pathology, Guangdong Provincial People’s Hospital, Guangzhou, Guangdong China; 5 Department of Pathology, Qilu Hospital of Shandong University, Shandong, China; 6 Department of Pathology, Guangdong Provincial Hospital of Chinese Medicine, Guangzhou, Guangdong China; 7 Department of Pathology, Union Hospital, Tongji Medical College, Huazhong University of Science and Technology, Wuhan, Hubei China; 8 Department of Pathology, Henan Provincial People’s Hospital, Henan, China; 9 Department of Pathology, The First Affiliated Hospital of Xi’an Jiaotong University, Shaanxi, China; 10 Department of Pathology, Sun Yat-sen Memorial Hospital, Sun Yat-sen University, Guangzhou, Guangdong China

**Keywords:** Breast cancer, Concordance, HER2-low, HER2-ultralow, Prevalence

## Abstract

**Background:**

Accurately assessing HER2-low (immunohistochemistry [IHC] 1 + and IHC 2+/in situ hybridization [ISH]–) and HER2-ultralow (IHC > 0 < 1+) is essential given the emergence of novel therapies. Thorough understanding of the reproducibility of rescoring IHC stained slides or re-staining archived tissue slides is essential.

**Methods:**

2,869 breast cancer patients diagnosed between July 2021 and July 2022 from 10 hospitals in China were included in this multicentre study. The prevalence of different HER2 expression levels and distribution of HER2 IHC scores were assessed by HER2 status determination from rescored historical slides. Concordance was evaluated across historical results versus rescored results, historical results versus re-stained results, and leading center results versus local site results. Clinicopathological characteristics were retrospectively analyzed as well.

**Results:**

HER2 IHC 0, IHC 1+, IHC 2+, and IHC 3 + were identified in 682 (23.8%), 871 (30.4%), 801 (27.9%), and 515 (18.0%) cases, respectively. HER2-positive, HER2-low, and HER2 IHC 0 (HER2-ultralow and IHC null) were identified in 21.7%, 54.5%, and 23.8% of cases, respectively. The prevalence of HER2-ultralow and IHC null was 10.6% and 13.2%, respectively. The concordance for HER2-ultralow was 43.3%; 30% of cases that were scored as HER2-ultralow at local sites were rescored as HER2-null and 26.7% of cases were rescored as IHC 1 + at the leading site. Overall, there was substantial agreement (83.1%) between rescored and historical IHC results. A high concordance rate of 91.7% was observed for HER2-low classification.

**Conclusions:**

This is the first multicenter study to determine the prevalence of HER2-low and HER2-ultralow based on rescored results in the Chinese breast cancer population. The concordance analysis carries important implications for the diagnosis of HER2-low and HER2-ultralow cases in clinical practice. The relatively low concordance in identifying HER2-ultralow suggested that the reproducibility of scoring HER2-ultralow needed to be improved through training.

**Trial registration:**

ClinicalTrials.gov identifier NCT05203458.

**Supplementary Information:**

The online version contains supplementary material available at 10.1186/s13058-025-02001-0.

## Background

Breast cancer is the most common malignancy affecting women worldwide, with approximately 2.3 million new cases diagnosed each year [1]. The overexpression of the *HER2* gene, found in approximately 15–20% of patients with metastatic breast cancer, is associated with a high risk of recurrence and poor prognosis [2]. Traditionally, the determination of HER2 status has been a binary classification, dividing patients into HER2-positive (immunohistochemistry [IHC] scores of 3 + or IHC 2 + with a positive in situ hybridization [ISH+]) and HER2-negative (IHC scores of 0, 1+, or 2 + with a negative ISH [ISH–]) categories. This classification plays a crucial role in guiding therapeutic strategies and has been widely adopted in clinical practice [3, 4]. In recent years, HER2-low breast cancer, characterized by HER2 IHC scores of 1 + or 2 + along with a ISH- result, has become eligible for treatment with trastuzumab deruxtecan (T-DXd) based on the results of the DESTINY-Breast04 trial [5]. Patients with HER2-low breast cancer are now recognized as being part of a distinct subgroup who may benefit from T-DXd, and this was affirmed in a 2023 update of the American Society of Clinical Oncology (ASCO-CAP) guidelines [6]. This changes the conventional dichotomous classification of HER2 expression and, thus, our understanding and management of breast cancer based on HER2 expression level. The lowest threshold of HER2 expression via IHC that is indicative of benefit from HER2-directed antibody-drug conjugates remains to be defined. Results from the DESTINY-Breast06 trial demonstrated that not only patients with HER2-low breast cancer but also those with HER2-ultralow disease (defined as IHC 0 with incomplete and faint staining in ≤ 10% of tumor cells) benefited from T-DXd, suggesting that identifying HER2-ultralow disease is also clinically relevant [7–9]. This has led to the approval of T-DXd for metastatic HER2-low or HER2-ultralow breast cancer by the US Food and Drug Administration [9, 10].

Understanding the precise prevalence of the HER2-low breast cancer subtype in different patient populations is crucial because of its therapeutic implications. Recent estimates suggest that HER2-low represents a large majority of HER2-negative breast cancer tumors [11–15]. In several multicenter, international studies, 60–67% of HER2-negative patients with breast cancer were classified as having HER2-low disease [11, 12]. However, reports on the prevalence of HER2-low expression in Chinese patients are limited [13–15], and most of the available data are based on historical results, which primarily aimed to identify the HER2-positive populations [12–16]. As a result, high-quality data on the prevalence of HER2-low and HER2-ultralow expression are lacking in Chinese patients with breast cancer.

With HER2-low breast cancer emerging as a new targetable subset, it is imperative to ensure accurate and consistent diagnosis of HER2-low status, which serves the foundation for guiding HER2-directed treatment decisions. Although IHC and ISH remain the primary methods for identifying patients with HER2-low status, it should be noted that historical scoring methods have primarily focused on identifying HER2-positive populations. A multicenter, worldwide study by Viale et al. showed that a significant proportion of cases with historical score of 0 were reclassified as 1 + upon re-evaluation [11]. Recent research by Fernandez et al. has highlighted the poor scoring accuracy for HER2 IHC in the range of 0 to 1 + based on the CAP survey data set, where the concordance between HER2 0 and 1 + was 26.0% and 2 + and 3 + was 58.0% [17]. Several other studies have also reported notable interobserver variations, ranging from fair to substantial agreement in the assessment of HER2-low breast cancer, with lower agreement frequently observed in cases with scores of 0–1+ [12, 17, 18].

The current retrospective study sought to address the aforementioned questions. First, based on the rescored results, this study will provide a reliable estimation of HER2-low and HER2-ultralow prevalence in patients with breast cancer in China. Second, as interobserver variation continues to be a challenge in IHC testing, especially for IHC 0 and 1+, evaluation of concordance rates would confirm whether the results can be interpreted with reasonable confidence and used reliably in the diagnosis of HER2-low disease [11, 17, 19].

## Methods

### Study design

This was a multicenter, retrospective study (HER2-PATH, NCT05203458) in patients with confirmed diagnosis of breast cancer in China. The primary objective was to accurately assess the prevalence of different levels of HER2 expression and distribution of HER2 IHC scores in the 2,869-patient cohort. Secondary objectives included the concordance rates of historical results versus rescore results, historical results versus re-stained or rescored results, and leading center results versus local site results. Eligible patients were aged ≥ 18 years, had histological confirmed diagnosis of breast cancer between July 2021 and July 2022, had ≥ 1 archived HER2 IHC slide in good condition for rescoring, and had available fluorescence in situ hybridization (FISH) results for HER2 IHC2+. The detailed exclusion criteria are listed in Additional File 1.

### Study flow and assessment

**Fig. 1:**
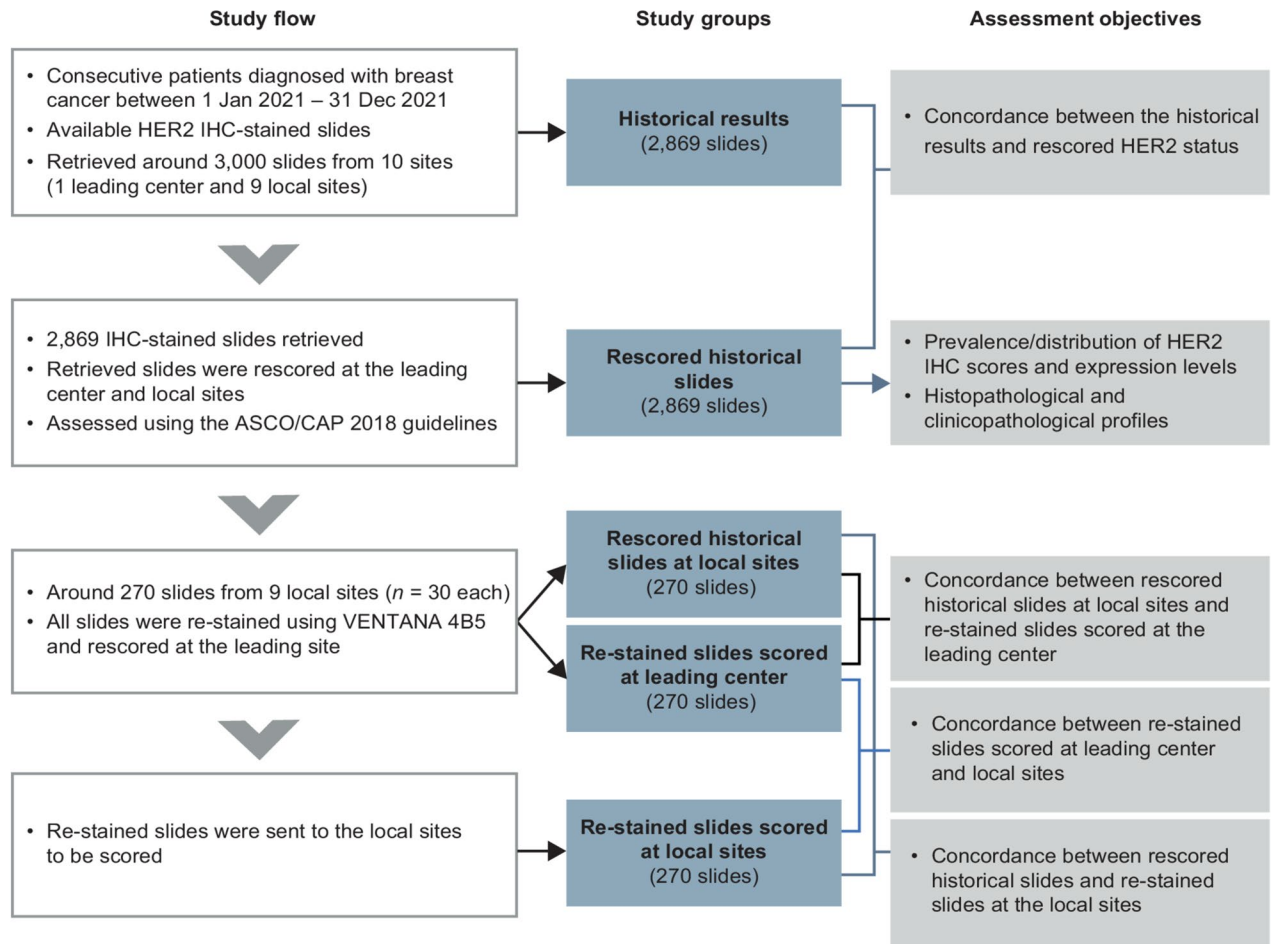
Study design

The study flow and assessment groups are illustrated in Fig. 1. Patients from 10 medical centers in China who underwent breast cancer surgery between July 2021 and July 2022 were included in the study. A total of 300 patients per site were included chronologically, with Fudan University Shanghai Cancer Center (FUSCC) being the leading study center. Relevant information, including patients’ general details, demographic data, diagnosis, clinicopathological features, and historical HER2 IHC scores obtained by VENTANA HER2 (4B5) Assay were extracted from medical records. Archived HER2 IHC slides (tissue sample obtained via surgery) from these patients were subjected to rescoring by a review committee, who were blinded to the historical results. The HER2 scoring scheme and nomenclature are presented in Additional File 1: Fig. S1. At the beginning of this multicentre study, pathologists from all participating sites underwent training and were aligned on the scoring criteria. The major recommendations for interpretation training included magnification rules and scoring framework (intensity, membrane completeness, and percentage). The training also included case studies highlighting pitfalls in HER2 interpretation, as well as examples of special staining patterns and nonspecific staining.

### Re-staining and rescoring at FUSCC

A total of 270 patient samples from 9 local sites (30 samples per site) were chosen for HER2 IHC re-staining. Within each site, a random selection process was used to choose 8 patients with a HER2 IHC score of 0, 9 patients with a score of 1+, 9 patients with a score of 2+, and 4 patients with a score of 3+. These selected samples were then resectioned and sent to FUSCC for re-staining and rescoring. Subsequently, the re-stained slides were returned to their respective centers for scoring by the local review committee independently.

All slides were stained using the VENTANA HER2 (4B5) Assay and scored following the ASCO/CAP 2018 guidelines, including the addition of HER2-ultralow as defined in the DESTINY-Breast 06 trial.

### Review committee

The pathologists committee consisted of 3 professional pathologists at each site: 2 to read the set of 30 cases, and 1 to serve as adjudicator for discrepant cases. Each of the 2 readers independently evaluated the same 30 samples. If the results between the 2 readers matched, it was recorded as the final result. Otherwise, the adjudicator would review each reader’s scores before making the final judgement on HER2 IHC status.

### Statistical analysis

The prevalence of different HER2 expression levels was calculated based on the rescored HER2 status. The corresponding 95% confidence interval (CI) was calculated using the Clopper-Pearson’s exact method. For concordance analysis between historical and rescored IHC scores or between different sites, the shift table with count and percentage was provided to summarize the distribution of each circumstance of disagreement. The overall agreement was examined using the Cohen’s Kappa coefficient, whereby convention, a Kappa value equal to or greater than 0.8, is often considered almost perfect agreement, and a Kappa value between 0.8 and 0.6 is considered substantial agreement. All statistical procedures were performed using SAS v.9.4.

### Ethics

This study was approved by the ethics committees of the study sites and performed in accordance with the Declaration of Helsinki, International Council for Harmonisation for Good Clinical Practice, and the applicable legislation on non-interventional studies and/or observational studies. No informed consent was needed in this retrospective study.

## Results

### Baseline demographic and characteristics

A total of 2936 patients were screened, of whom 2869 with IHC-stained slides were included in this study. All patients were Chinese, the majority were female (*n* = 2855, 99.5%) and the median age was 52.8 years (range: 20.2–92.2 years) (Additional File 1: Table S1). A total of 22.8% (*n* = 654) of historical HER2 IHC scores were 0, 30.6% (*n* = 877) were 1+, 29.5% (*n* = 846) were 2+, and 17.1% (*n* = 492) were 3+. Among IHC 2 + patients, 85.0% (*n* = 719) were FISH– and 15.0% (*n* = 127) were FISH+. The proportions of HER2 expression level based on historical assessment were 22.8% (*n* = 654) for HER2 IHC 0, 55.6% (*n* = 1596) for HER2-low, and 21.6% (*n* = 619) for HER2-positive (Additional File 1: Table S1). As for hormone receptor (HR) status, 2277 (79.4%) were HR-positive and 591 (20.6%) were HR-negative.

### Prevalence

**Fig. 2:**
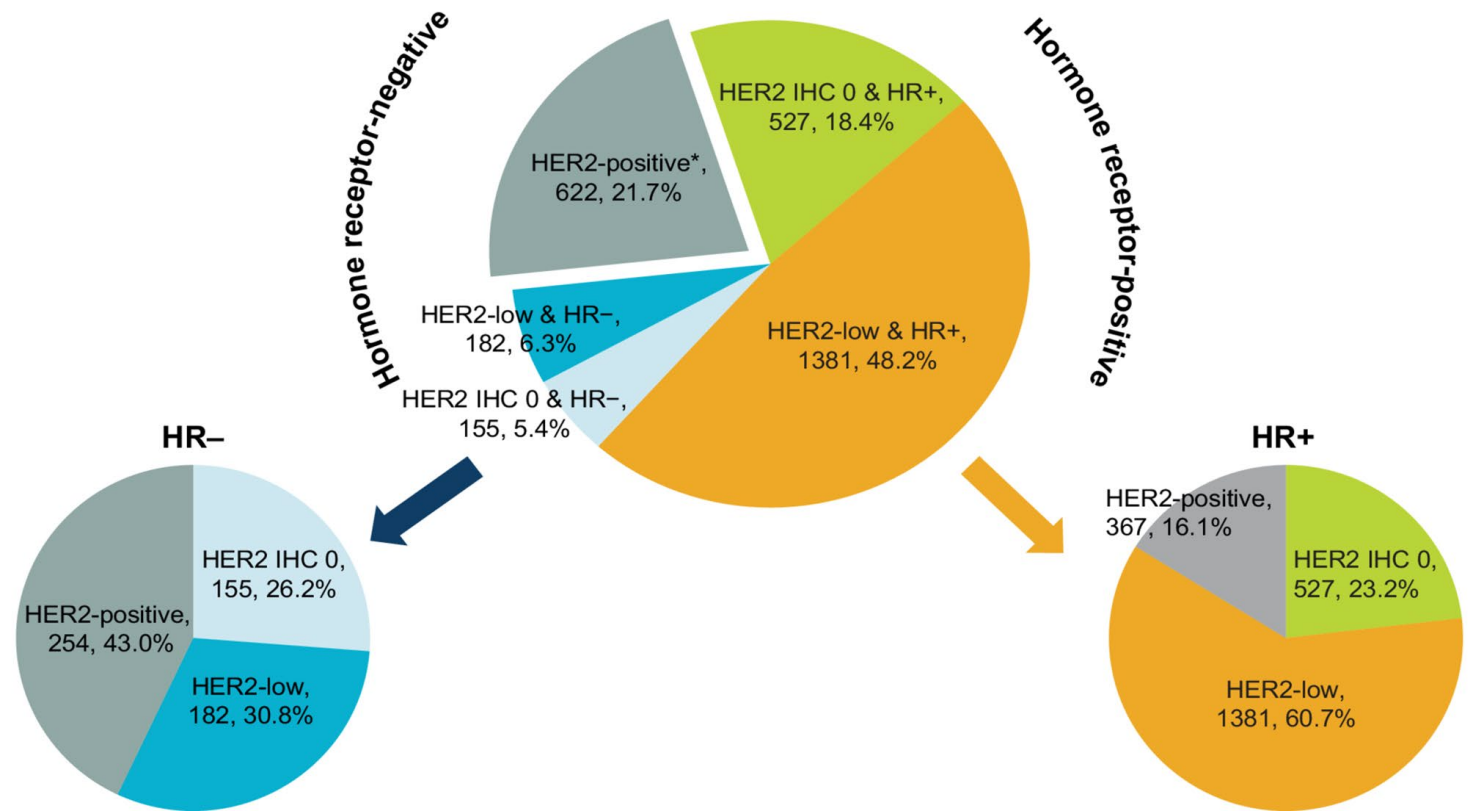
Distribution of HER2 expression among HR positive and HR negative subgroups from rescored historical slides. HER2 IHC 0 is defined as HER2 null and HER2-ultralow; HER2-low is defined as IHC 1 + or IHC 2+/ISH-; HER2-positive is defined as IHC 3 + or IHC 2+/ISH+. HER2 null indicates IHC 0 with no staining. * HR status was missing for one patient

Based on the rescored results conducted by the pathologist committee, 682 patients (23.8%) were classified as IHC 0, 871 (30.4%) as IHC 1+, 801 (27.9%) as IHC 2+, and 515 (18.0%) as IHC 3+ (Table 1). Among all patients, prevalences of HER2-ultralow IHC > 0 to < 1 + and HER2 null were 10.6% (*n* = 379) and 13.2% (*n* = 303), respectively. When the FISH results were combined, the prevalence of HER2-low was 54.5% (95% CI, 52.7–56.3%). The distribution of HER2 expression stratified by HR status was analyzed. HER2-low prevalence was higher in the HR-positive than HR-negative subgroup (60.7% vs. 30.8%) (Fig. 2). The prevalence of HER2-ultralow was 10.9% and 9.1% in the HR-positive and HR-negative subgroups, respectively.

**Table 1:** Distribution of HER2 IHC score, FISH results, and HER2 expression levels from rescored historical slides

Characteristics	Total patients (*N* = 2869)
HER2 IHC score, *n* (%)	
0	682 (23.8%)
Null	379 (13.2%)
Ultralow	303 (10.6%)
1+	871 (30.4%)
2+	801 (27.9%)
3+	515 (18.0%)
Total	2869
FISH results for HER2 IHC 2 + cases, *n* (%)	
FISH-	692 (86.6%)
FISH+	107 (13.4%)
Missing	2
Total	801
HER2 expression level based on the rescored HER2 status, *n* (%)	
HER2 IHC 0	682 (23.8%)
HER2 null	379 (13.2%)
HER2-ultralow	303 (10.6%)
HER2-low	1563 (54.5%)
HER2-positive	622 (21.7%)
Missing	2
Total	2869

HER2 expression level: (a) HER2 IHC 0: defined as HER2 null or HER2-ultralow; (b) HER2-low: defined as IHC 1 + or IHC 2+/FISH–; (c) HER2-positive: defined as IHC 2+/FISH + or IHC 3+; (d) Missing: missing rescored HER2 status, or IHC 2 + with unknown or missing FISH result

FISH, fluorescence in situ hybridization

### Concordance

#### Concordance of historical results versus rescored slides

The concordance between historical scores and rescores of the same slides for HER2 IHC score was 83.1% (2383/2869) (Table 2). Overall, there was substantial agreement between both groups for IHC scores (κ = 0.77; 95% CI, 0.75–0.79). A lower concordance rate was seen in the group with HER2 IHC 1+ (74.5%) compared with those with IHC 0 (85.2%), IHC 2+ (81.3%), and IHC 3+ (98.6%). 14.7% (*n* = 96) of IHC 0 cases from the historical slides were rescored to IHC 1+, and 13.5% (*n* = 118) of IHC 1 + were rescored to IHC 0; 12.1% (*n* = 106) of IHC 1 + cases were rescored to IHC 2+. Most discordant cases occurred between IHC 0 versus IHC 1+ (*n* = 214) and IHC 1 + versus IHC 2+ (*n* = 227).

**Table 2:** Concordance of HER2 IHC score and expression level between historical results and rescored historical slides

HER2 IHC score	Historical results							
	0	1+		2+		3+	Total	
Rescored historical slides								
0	557 (85.2%)	118 (13.5%)		7 (0.8%)		0 (0.0%)	682 (23.8%)	
Null*	347 (53.1%)	28 (3.2%)		4 (0.5%)		0 (0.0%)	379 (13.2%)	
Ultralow*	210 (32.1%)	90 (10.3%)		3 (0.4%)		0 (0.0%)	303 (10.6%)	
1+	96 (14.7%)	653 (74.5%)		121 (14.3%)		1 (0.2%)	871 (30.4%)	
2+	1 (0.2%)	106 (12.1%)		688 (81.3%)		6 (1.2%)	801 (27.9%)	
3+	0 (0.0%)	0 (0.0%)		30 (3.5%)		485 (98.6%)	515 (18.0%)	
Total	654	877		846		492	2869	
Agreement	557	653		688		485	2383 (83.1%)	
Kappa coefficient and 95% CI							0.77 (0.75–0.79)	

**HER2 expression level**	**Historical results**							
	**HER2 IHC 0**		**HER2-low**		**HER2-positive**		**Total**	
Rescored historical slides								
HER2 IHC 0	557 (85.2%)		125 (7.8%)		0 (0.0%)		682 (23.8%)	
HER2 null*	347 (53.1%)		32 (2.0%)		0 (0.0%)		379 (13.2%)	
HER2-ultralow*	210 (32.1%)		93 (5.8%)		0 (0.0%)		303 (10.6%)	
HER2-low	97 (14.8%)		1461 (91.7%)		5 (0.8%)		1563 (54.5%)	
HER2-positive	0 (0.0%)		8 (0.5%)		614 (99.2%)		622 (21.7%)	
Missing	0		2		0		2	
Total	654		1596		619		2869	
Agreement	557		1461		614		2632 (91.7%)	
Kappa coefficient and 95% CI							0.86 (0.85–0.88)	

Note: the top half of the table computes the concordance of HER2 IHC score between rescored historical slides and historical results; the bottom half of the table computes the concordance of HER2 expression level of the rescored historical slides and historical results

Total refers to the sum of all patients in each column or row; agreement refers to the total number of patients with concordant results; Kappa coefficient refers to the overall agreement between historical results and rescoring. The denominator of the percentage calculation is the number of total patients or subgroup. *HER2 null and HER2 ultralow subgroups were not used to calculate agreement/concordance

The overall concordance for HER2 expression status was 91.7% (2632/2869). The highest concordance was achieved with HER2-positive samples at 99.2%, while those for HER2 IHC 0 and HER2-low were 85.2% and 91.7%, respectively (Table 2). A κ value of 0.86 (95% CI, 0.85–0.88) indicated almost perfect agreement for HER2 expression status between the historical results and rescored results. 7.8% (*n* = 125) of HER2-low cases from the historical results were reassigned to HER2 IHC 0 and 14.8% (*n* = 97) of HER2 IHC 0 were reassigned to HER2-low after rescoring. A smaller proportion (0.5% [*n* = 8]) was reassigned from HER2-low to HER2-positive.

#### Concordance of re-stained slides scored at the leading center versus local sites

The concordance according to the HER2 IHC category for the re-stained slides scored at the leading center and local sites was 82.2% (222/270, Table 3). The κ value was 0.77 (95% CI, 0.70–0.83) indicating substantial agreement. Most discordant cases occurred between HER2 IHC 0 versus 1+ (*n* = 20) and 1 + versus 2+ (*n* = 23) categories. However, this had limited impact on the HER2 status as the overall κ concordance score was 0.88, which is considered almost perfect agreement. After adjusting for extreme bias (such as IHC 1 + to 3 + and vice versa), the quadratic weighted κ was 0.85 for IHC category and 0.93 for HER2 expression level between the leading center and the local sites. Additionally, the κ values between the leading center and each local site for HER2 IHC category ranged from 0.66 to 0.86 and 0.83 to 0.95 for HER2 expression status, suggesting substantial to almost perfect agreement (Additional File 1: Tables S2 and S3). Moreover, the concordance for HER2-ultralow was only 43.3%, while 30% of cases scored as HER2-ultralow at local sites were rescored as HER2-null and 26.7% of cases were rescored as IHC 1 + at the leading site (Additional File 1: Table S4).

**Table 3:** Concordance between re-stained slides scored at leading center and local sites

HER2 IHC score		Re-stained slides scored at local sites																		
		0			Null*		Ultralow*		1+			2+			3+		Total			
**Re-stained slides scored at leading center**																				
0		89 (89.9%)			67 (97.1%)		22 (73.3%)		10 (13.5%)			0 (0.0%)			0 (0.0%)		100 (37.2%)			
Null*		62 (62.6%)			53 (76.8%)		9 (30.0%)		2 (2.7%)			0 (0.0%)			0 (0.0%)		65 (24.2%)			
Ultralow*		27 (27.3%)			14 (20.3%)		13 (43.3%)		8 (10.8%)			0 (0.0%)			0 (0.0%)		35 (13.0%)			
1+		10 (10.1%)			2 (2.9%)		8 (26.7%)		60 (81.1%)			19 (33.3%)			0 (0.0%)		89 (33.1%)			
2+		0 (0.0%)			0 (0.0%)		0 (0.0%)		4 (5.4%)			37 (64.9%)			1 (2.7%)		43 (16.0%)			
3+		0 (0.0%)			0 (0.0%)		0 (0.0%)		0 (0.0%)			1 (1.8%)			36 (97.3%)		37 (13.8%)			
Missing		0			0		0		0			0			0		1			
Total		99			69		30		74			57			37		270			
Agreement		89			53		13		60			37			36		222 (82.2%)			
Kappa coefficient (95% CI)																	0.77 (0.70–0.83)			
Quadratic weighted Kappa coefficient (95% CI)																	0.85 (0.81–0.89)			

**HER2 expression level**	**Re-stained slides scored at local sites**																			
	**HER2 IHC 0**					HER2 null*		HER2-ultralow*			**HER2-low**			**HER2-positive**				**Total**		
**Re-stained slides scored at leading center**																				
HER2 IHC 0	89 (89.9%)					67 (97.1%)		22 (73.3%)			10 (8.6%)			0 (0.0%)				100 (37.2%)		
HER2 null*	62 (62.6%)					53 (76.8%)		9 (30.0%)			2 (1.7%)			0 (0.0%)				65 (24.2%)		
HER2-ultralow*	27 (27.3%)					14 (20.3%)		13 (43.3%)			8 (6.9%)			0 (0.0%)				35 (13.0%)		
HER2-low	10 (10.1%)					2 (2.9%)		8 (26.7%)			106 (91.4%)			0 (0.0%)				117 (43.5%)		
HER2-positive	0 (0.0%)					0 (0.0%)		0 (0.0%)			0 (0.0%)			52 (100.0%)				52 (19.3%)		
Missing	0					0		0			0			0				1		
Total	99					69		30			116			52				270		
Agreement	89					53		13			106			52				247 (91.5%)		
Kappa coefficient (95% CI)																		0.88 (0.83, 0.93)		
Quadratic weighted Kappa coefficient (95% CI)																		0.93 (0.90, 0.96)		

Note: the top half of the table computes the concordance of HER2 IHC score of the re-stained slides scored at the leading center versus at the local sites; the bottom half of the table computes the concordance of HER2 expression level of the re-stained slides scored at the leading center versus at the local sites

Total refers to the sum of all patients in each column or row; agreement refers to the total number of patients with concordant results; Kappa coefficient refers to the overall agreement between leading center and local sites for the re-stained slides; quadratic weighted Kappa coefficient takes into consideration of the levels of disagreement between groups. The denominator of the percentage calculation is the number of total patients or subgroup. *HER2 null and HER2-ultralow subgroups were not used to calculate Kappa coefficients

#### Concordance of re-stained versus historical slides both scored at local sites

The overall concordance for HER2 IHC category between historical and re-stained slides assessed at the local sites was 71.5% (193/270; Additional File 1: Table S5). The κ value was 0.62, which suggests substantial agreement. Most discordant cases occurred between HER2 IHC 0 versus 1+ (*n* = 41) and IHC 1 + versus 2+ (*n* = 27). Regarding HER2 status, the κ concordance score was 0.73 and most discordant cases occurred between HER2 IHC 0 and HER2-low (*n* = 44). The κ values between both groups ranged from 0.50 to 0.86 for HER2 IHC category, indicating moderate to almost perfect agreement among local sites (Additional File 1: Table S2), and from 0.62 to 0.90 for HER2 expression status, indicating substantial to almost perfect agreement (Additional File 1: Table S3).

#### Concordance of re-stained versus historical slides, scored at leading center and local sites, respectively

We also compared the concordance between re-stained slides scored at the leading center and historical slides rescored at the local sites. The overall concordance rate was 68.5% (185/270; Additional File 1: Table S6), and κ value was 0.57 indicating moderate agreement. Most discordant cases also occurred between IHC 0 versus 1+ (*n* = 38) and 1 + versus 2+ (*n* = 37). This has limited impact on HER2 status as the κ value of 0.74 indicates substantial agreement. There were 42 discordant cases occurring between HER2 IHC 0 and HER2-low. Overall, the κ values between both groups ranged from 0.29 to 0.91, suggesting fair to almost perfect agreement (Additional File 1: Table S2), and from 0.53 to 0.95 for HER2 expression status, indicating moderate to perfect agreement (Additional File 1: Table S3).

### Clinicopathological characteristics

The clinicopathological characteristics of patients rescored at the leading center are listed in Additional File 1: Table S7. In general, the clinicopathological profiles were similar for HER2-low and HER2 IHC 0 disease, with no notable differentiating features.

## Discussion

HER2 is a predictive biomarker to guide treatment decision and prognosis in breast cancer. Recent emerging evidence has highlighted the clinical significance of distinguishing between HER2 IHC score 0 and 1 + in patients with HER2-low disease. To ensure accurate categorization and avoid misdiagnosis of breast cancer patients for decision making, it is necessary to assess whether current testing methods can identify HER2-low disease with reasonable reproducibility.

To our knowledge, this study is the first large multicenter study conducted in China to assess the prevalence and concordance of HER2 status among all patients with breast cancer (including HER2-postive and HER2-negative) based on rescored results using an expert panel. We found that the prevalence of HER2-low and HER2-ultralow was 54.5% and 10.6%, respectively, among a cohort of 2869 patients with breast cancer, suggesting that approximately half of all breast cancer patients may benefit from HER2-low targeted treatments. Notably, we observed that 14.8% of patients initially classified as HER2 IHC 0 were reassigned to HER2-low after rescoring at the leading center, suggesting that more attention should be given to these patients during diagnosis, and signaling that this group could be rescored to determine treatment eligibility with T-DXd. The HER2-low status in our study was more prevalent in the HR-positive group than the HR-negative subgroup (60.7% vs. 30.8%), which is consistent with previous studies [11, 20].

The prevalence of HER2-low cancer in China is well described in the literature, especially for HER2-negative breast cancer [13–15]. One study found HER2-low in 59.2% of 5610 consecutive patients with early-stage breast cancer [21]. Another study reported HER2-low disease in 48.5% of 1,250 female patients with primary non-metastatic breast cancer [16]. Dai et al., using rescored historical HER2 IHC slides, found HER2-low disease in 61.3% of 707 consecutive patients who underwent breast cancer surgery. However, most of these studies utilized historical HER2 IHC scores and included patients diagnosed with breast cancer between 2000 and 2010, when awareness of HER2-low disease was limited [13–16, 21]. Various factors, such as missing histological or IHC information, changing HER2 definitions and scoring criteria over time, and the lack of treatment options and voluntary FISH testing in the past, may have impacted the reported prevalence [12, 13, 15, 22]. The prevalence of HER2-low disease stratified by HR subgroups in our study aligns with that in previous studies (40–71% in HR-positive tumors; 27–53% in HR-negative tumors) [11, 12, 20, 22, 23]. However, four of these studies only examined HER2-negative breast cancers [11, 12, 22, 23], and only one included all breast cancer patients [20].

An important aspect of our study was the assessment of the population of patients with HER2-ultralow disease (defined as IHC 0 with incomplete and faint staining in ≤ 10% of tumor cells), a population currently being investigated in DESTINY-Breast06 with T-DXd [24]. We found that HER2-ultralow tumors accounted for 10.6% of all patients with breast cancer in our study, slightly lower than reported rates in Germany (15.9%) and in the United Kingdom and Ireland (12.0%) [18, 25]. The relatively low concordance (43.3%) in identifying HER2-ultralow between the leading site and local sites suggested that the reproducibility of HER2 -ultralow scoring was relatively poor and required improvement through training. Concordance was also assessed among those with valid central and local HER2 test results in DESTINY-Breast06 trial. Of the 349 samples scored as IHC 0 locally (separating HER2-ultralow from IHC 0 was not standard practice at the local sites), central tests found that 40% were HER2-ultralow and 24% were HER2-low. Given that the results of this study showed T-DXd improved progression-free survival versus physician choice of chemotherapy in both the HER2-low and HER2-ultralow groups [8, 9], consistent across the different IHC scores (IHC 0 with membrane staining, 1+, and IHC2+/ISH–) [24], further highlighting that a considerable proportion of patients with a history of IHC 0 status could benefit from T-DXd treatment. Consequently, there is a need for increased awareness of HER2-low and -ultralow expression levels in clinical practice.

The overall concordance between historical results and rescored samples was 91.7% for HER2 status, with κ value indicating almost perfect agreement. This signaled that most cases could be reproducibly classified using archived stained slides, providing added confidence that HER2 status can be assigned reliably. However, a lower concordance rate of 83.1% was observed for some IHC categories, particularly in distinguishing HER2 IHC 0, 1+, and 2+. There was a substantial proportion of historical IHC 1 + that was rescored as IHC 2 + and vice versa, although this had limited impact on the assignment of HER2-low status as the concordance for HER2-low expression remained high. Poor distinction between IHC 0 and 1 + could potentially have negative clinical implications.

Interobserver variation is well documented when evaluating HER2 IHC scores, especially in cases with scores of 0 to 1+ [17–19, 26, 27]. We re-stained archived tissue slides at the central site before sending them to the respective local sites for scoring. The overall concordance for HER2 IHC categories was 82.2%, with the κ value suggesting substantial agreement. The agreement for HER2-low between central and local sites for re-stained slides was 91.4% (Table 3). However, the agreement between re-stained slides that were centrally scored, and historical slides rescored at local sites was lower at 75.3%. This was comparable with the 77.8% overall agreement for HER2-low reported in the DESTINY-Breast06 study [24]. A considerable number of discordant cases were observed for IHC 0 versus 1 + and 1 + versus 2+. However, these discrepancies had limited impact on HER2 status, with κ value suggesting almost perfect agreement. Importantly, there was diagnostic consistency between the leading center and local sites in our study. Previous studies have shown that standard training may improve the concordance of HER2-low diagnosis. In one such study, pathologists who completed a 4-hour structured training program, consisting of lectures, didactic microscope sessions, and case discussions, demonstrated improved proficiency in distinguishing between HER2 0 from HER2-low cases [28]. At the beginning of this study, pathologists were trained and aligned on the scoring criteria at all sites, thereby contributing to the high concordance observed for HER2-low cases compared with other studies. However, we also found that not all local sites showed perfect concordance with the leading site, underscoring the need to further strengthen training in the diagnosis of HER2-low breast cancer. Furthermore, the concordance for HER2-ultralow was relatively lower, indicating that pathologists may interpret HER2-ultralow criteria inconsistently. Given the clinical relevance of this subgroup, as evidenced by the positive results of the DESTINY-Breast06 trial, future efforts should prioritize training on the diagnosis of HER2-ultralow breast cancer [8]. Such training could include the reading of IHC-stained slides, with a focus on identifying staining patterns that are frequently observed in weakly stained samples. It could also cover interpretation techniques, particularly for cases bordering on the cut-off between HER2-low, -ultralow, and IHC 0 classifications [28].

Another interesting finding was that re-staining archived tissue samples may not always be a suitable approach to assess HER2 IHC scores, as evidenced by the poor concordance between the re-stained slides and rescored archived slides assessed at the local sites (overall concordance: 71.5%; κ value: 0.62). The level of agreement was also lower when comparing the re-stained slides scored at the leading center with the historical slides scored at the local sites (overall concordance: 68.5%; κ value: 0.57). The study was not able to control the staining protocol used for each laboratory, which might affect the concordance rate. Interobserver variation may exist between the leading center and local sites when a different set of slides was used (re-stained vs. historical) as indicated by the wide range for κ concordance values (0.29–0.91) (Additional File 1: Table S3). Further investigation would be needed to elucidate other potential factors that could affect the staining of archived paraffin-embedded tissues.

Our study has several important implications for pathology laboratories. First, rescoring archived IHC-stained slides is a reasonable method for assessing HER2 IHC scores and reassigning HER2 status, given the high level of agreement with historical results. This approach may spare some patients from invasive rebiopsy and avoid challenges such as limited tissue availability and increased patient burden in the hospital setting. However, it is worth noting that it is still not recommended to rescore archived IHC slides that were stained for too long. Second, our findings highlight significant interobserver variation when distinguishing HER2 IHC 0, 1+, and 2+. The variability may be attributed to subjective interpretation by individual pathologists or differences in experience levels. To address this, measures such as additional HER2-low training, establishing a consensus among readers for HER2 IHC evaluation, rigorous quality control procedures, adherence to guidelines, and increased awareness among pathologists on the importance of accurately quantifying lower levels of HER2 expression IHC can be implemented [27, 29]. It should be noted that in the study HER2 status was evaluated in surgical resection specimens. Core needle biopsy is often used in practice due to its convenience to provide diagnostic results before the surgical procedure [30]. The concordance of HER2-low status between core needle biopsy and surgical resection has been explored in several studies, with varying results ranging from low to moderate concordance [31–33].

Our study has several strengths. To our knowledge, it was the first study with a large patient population to evaluate the prevalence of HER2-low and HER2-ultralow in patients with breast cancer in China with all HER2 status through rescoring of archived IHC-stained slides. Unlike many other studies that only included patients classified as HER2-negative, we consecutively enrolled all breast cancer patients between July 2021 and July 2022 from 10 study sites, providing a more accurate estimation of HER2-low prevalence. Our study also has several limitations. The prevalence of HER2-positive breast cancer may be underestimated in our study as we excluded those who received neoadjuvant therapy. Moreover, the patient samples retrieved from 10 study sites may not be representative of the general population in China. There was also no external validation by an independent team or a panel of pathologists with varying levels of proficiency, which was a limitation of the study. The study also did not conduct or provide genomic or biological experiments or explanations, nor examine the correlation between HER2 status and clinical outcomes.

## Conclusions

In conclusion, our study showed that approximately 54.5% of patients with breast cancer in China had HER2-low disease and 10.6% of patients were HER2-ultralow. There was high concordance between historical and rescored groups, indicating that using archived IHC-stained slides as a means of identifying HER2-low patients is a reliable alternative to re-biopsy. Although some interobserver variability was identified, the agreement level for HER2 status was generally high between the leading center and local sites, implying that HER2 assessment could be reliably performed at all centers.

## Electronic supplementary material

Below is the link to the electronic supplementary material.

**Supplementary Material 1**: **Additional file 1**: **Table S1**: Baseline demographics and characteristics. **Table S2.** Kappa concordance value by individual local sites for HER2 IHC category. **Table S3.** Kappa concordance value by individual local sites for HER2 expression status. **Table S4.** Concordance between the leading center and the local sites on re-stained slides for HER2 expression level (including HER2-null and HER2-ultralow). **Table S5.** Concordance of historical slides and re-stained slides scored at the local sites for HER2 IHC category and HER2 expression level. **Table S6.** Concordance between re-stained slides scored at the leading center and historical slides rescored at the local sites for HER2 IHC category and HER2 expression level. **Table S7.** Histopathological and clinicopathological characteristics in the rescored/FUSCC group. **Figure S1**: HER2 scoring scheme and nomenclature.

## Data Availability

No datasets were generated or analysed during the current study.

## Abbreviations

ASCO: CAP american society of clinical oncology/college of american pathologists
CI: Confidence interval
FISH: Fluorescent in situ hybridization
FUSCC: Fudan university shanghai cancer center
HER2: Human epidermal growth factor receptor 2
HR: Hormone receptor
IHC: Immunohistochemistry
ISH: In situ hybridization
κ: Kappa
T-DXd: Trastuzumab deruxtecan
