# Supplementary material for: Prevalence and concordance of HER2-low and HER2-ultralow status between historical and rescored results in a multicentre study of breast cancer patients in China
Source: Breast Cancer Res. 2025 Mar 25;27:45. doi: 10.1186/s13058-025-02001-0 (PMC11934669; doi:10.1186/s13058-025-02001-0)
Supplement: Supplementary file 1 — Supplementary Material 1: Additional file 1: Table S1: Baseline demographics and characteristics. Table S2. Kappa concordance value by individual local sites for HER2 IHC category. Table S3. Kappa concordance value by individual local sites for HER2 expression status. Table S4. Concordance between the leading center and the local sites on re-stained slides for HER2 expression level (including HER2-null and HER2-ultralow). Table S5. Concordance of historical slides and re-stained slides scored at the local sites for HER2 IHC category and HER2 expression level. Table S6. Concordance between re-stained slides scored at the leading center and historical slides rescored at the local sites for HER2 IHC category and HER2 expression level. Table S7. Histopathological and clinicopathological characteristics in the rescored/FUSCC group. Figure S1: HER2 scoring scheme and nomenclature. [file 13058_2025_2001_MOESM1_ESM.docx]

**Additional Files 1**

**Prevalence and Concordance of HER2-Low and HER2-Ultralow Status Between Historical and Rescored Results in a Multicentre Study of Breast Cancer Patients in China**

**Hong Lv, et al.**

Item 5 of the inclusion criteria “at least one archived HER2 IHC slice related to the diagnosis of breast cancer, and the slices were in good condition for rescoring” is refined for the accuracy of the HER2 IHC scoring results and the final interpretation. Patients with the following conditions were excluded:

1. The sample information was incomplete and/or untraceable
2. Specimens collected by hollow needle puncture
3. Previously received any treatment (including neoadjuvant therapy)
4. Decalcified specimens
5. Microinvasive carcinoma
6. Frozen or frozen paraffin-embedded specimens
7. Specimens not preserved as required
8. Specimens with human errors in the testing process
9. The investigator’s judgement affected the interpretation of the specimen

# Figure S1**.** HER2 scoring scheme and nomenclature.


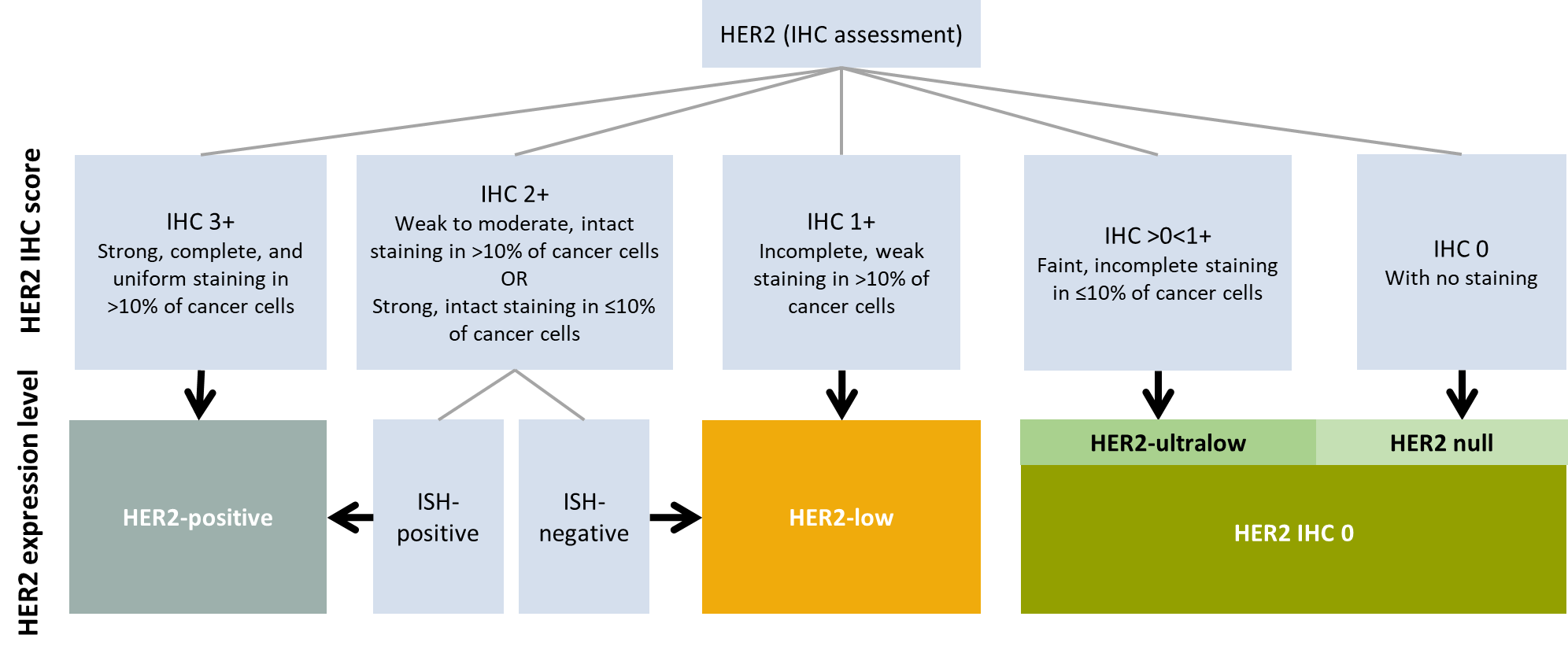


IHC, immunohistochemistry; ISH, in situ hybridization.

# Table S1. Baseline demographics and characteristics.

| **Characteristics** | **Total patients**  **(*N* = 2,869)** |
| --- | --- |
| Age at diagnosis (years) |  |
| *N* (Missing) | 2,869 (0) |
| Mean (SD) | 53.7 (11.55) |
| Median (P25, P75) | 52.8 (45.6, 61.7) |
| Min, Max | 20.2, 92.2 |
| Sex, *n* (%) |  |
| Male | 14 (0.5%) |
| Female | 2,855 (99.5%) |
| Total | 2,869 |
| Race, *n* (%) |  |
| Asian | 2,869 (100.0%) |
| Other | 0 (0.0%) |
| Total | 2,869 |
| Ethnicity, *n* (%) |  |
| Hispanic or Latino | 0 (0.0%) |
| Not Hispanic or Latino | 2,869 (100.0%) |
| Total | 2,869 |
| HER2 IHC historical scores, *n* (%) |  |
| 0 | 654 (22.8%) |
| 1+ | 877 (30.6%) |
| 2+ | 846 (29.5%) |
| 3+ | 492 (17.1%) |
| Total | 2,869 |
| FISH results for HER2 IHC historical scores with 2+, *n* (%) |  |
| FISH– | 719 (85.0%) |
| FISH+ | 127 (15.0%) |
| Total | 846 |
| HER2 expression level based on the historical HER2 status, *n* (%) |  |
| HER2 IHC 0 | 654 (22.8%) |
| HER2-low | 1,596 (55.6%) |
| HER2-positive | 619 (21.6%) |
| Total | 2,869 |

HER2 expression level: (a) HER2 IHC 0: defined as IHC null or IHC >0<1+; (b) HER2-low: defined as IHC 1+ or IHC 2+/FISH-; (c) HER2-positive: defined as IHC 2+/FISH+ or IHC 3+.

FISH, fluorescence in situ hybridization; IHC, immunohistochemistry; Max, maximum; Min, minimum; P25, 25th percentile; P75, 75th percentile; SD, standard deviation.

# Table S2**.** Kappa concordance value by individual local sites for HER2 IHC score.

|  | **Simple Kappa coefficient (95% CI)** | | |
| --- | --- | --- | --- |
| **Local site name** | **Leading center vs. local sites for re-stained slides** | **Re-stained slides scored at local sites vs. historical slides rescored at local sites** | **Re-stained slides** **scored at the leading center vs. historical slides rescored  at local sites** |
| **E1302** | 0.85 (0.70–1.00) | 0.55 (0.33–0.77) | 0.50 (0.27–0.73) |
| **E1303** | 0.86 (0.72–1.00) | 0.86 (0.72–1.00) | 0.91 (0.79–1.00) |
| **E1304** | 0.66 (0.45–0.87) | 0.68 (0.48–0.89) | 0.54 (0.33–0.76) |
| **E1305** | 0.85 (0.70–1.00) | 0.66 (0.43–0.88) | 0.82 (0.65–0.99) |
| **E1306** | 0.68 (0.46–0.90) | 0.58 (0.35–0.80) | 0.29 (0.07–0.52) |
| **E1307** | 0.70 (0.50–0.90) | 0.64 (0.43–0.85) | 0.46 (0.23–0.69) |
| **E1308** | 0.68 (0.46–0.89) | 0.54 (0.31–0.78) | 0.68 (0.47–0.89) |
| **E1309** | 0.86 (0.71–1.00) | 0.59 (0.36–0.82) | 0.59 (0.37–0.81) |
| **E1310** | 0.68 (0.48–0.89) | 0.50 (0.27–0.73) | 0.38 (0.15–0.60) |

The colors for each cell represent the following: green for almost perfect agreement (0.81–1), yellow for substantial agreement (0.61–0.8), orange for moderate agreement (0.41–0.6), and red for fair agreement (0.21–0.4).

CI, confidence interval.

# Table S3**.** Kappa concordance value by individual local sites for HER2 expression status.

|  | **Simple Kappa coefficient (95% CI)** | | |
| --- | --- | --- | --- |
| **Local site name** | **Leading center vs local sites for re-stained slides** | **Re-stained slides scored at local sites vs. historical slides rescored at local sites** | **Re-stained slides scored at the leading center vs. historical slides rescored  at local sites** |
| **E1302** | 0.95 (0.84–1.00) | 0.65 (0.43–0.87) | 0.70 (0.48–0.91) |
| **E1303** | 0.85 (0.69–1.00) | 0.90 (0.77–1.00) | 0.95 (0.85–1.00) |
| **E1304** | 0.84 (0.66–1.00) | 0.84 (0.66–1.00) | 0.89 (0.73–1.00) |
| **E1305** | 0.94 (0.82–1.00) | 0.70 (0.45–0.94) | 0.77 (0.56–0.98) |
| **E1306** | 0.83 (0.64–1.00) | 0.67 (0.44–0.90) | 0.53 (0.29–0.77) |
| **E1307** | 0.89 (0.74–1.00) | 0.68 (0.45–0.90) | 0.58 (0.35–0.82) |
| **E1308** | 0.83 (0.65–1.00) | 0.72 (0.49–0.95) | 0.89 (0.74–1.00) |
| **E1309** | 0.90 (0.75–1.00) | 0.74 (0.52–0.95) | 0.74 (0.52–0.95) |
| **E1310** | 0.89 (0.75–1.00) | 0.62 (0.37–0.86) | 0.63 (0.38–0.87) |

The colors for each cell represent the following: green for almost perfect agreement (0.81–1), yellow for substantial agreement (0.61–0.8), orange for moderate agreement (0.41–0.6), and red for fair agreement (0.21–0.4).

CI, confidence interval.

# Table S4**.** Concordance between the leading center and the local sites on re-stained slides for HER2 IHC score (including HER2-null and HER2-ultralow).

| HER2 IHC score | Re-stained slides scored at the local sites | | | | | | |
| --- | --- | --- | --- | --- | --- | --- | --- |
|  | **Null** | **Ultralow** | **1+** | **2+** | **3+** | **Missing** | **Total** |
| Re-stained slides scored at the leading center |  |  |  |  |  |  |  |
| Null | 53 (76.8%) | 9 (30.0%) | 2 (2.7%) | 0 (0.0%) | 0 (0.0%) | 1 | 65 |
| Ultralow | 14 (20.3%) | 13 (43.3%) | 8 (10.8%) | 0 (0.0%) | 0 (0.0%) | 0 | 35 |
| 1+ | 2 (2.9%) | 8 (26.7%) | 60 (81.1%) | 19 (33.3%) | 0 (0.0%) | 0 | 89 |
| 2+ | 0 (0.0%) | 0 (0.0%) | 4 (5.4%) | 37 (64.9%) | 1 (2.7%) | 1 | 43 |
| 3+ | 0 (0.0%) | 0 (0.0%) | 0 (0.0%) | 1 (1.8%) | 36 (97.3%) | 0 | 37 |
| Missing | 0 | 0 | 0 | 0 | 0 | 1 | 1 |
| Total | 69 | 30 | 74 | 57 | 37 | 3 | 270 |
| Agreement | 53 | 13 | 60 | 37 | 36 | – | 199 (73.7%) |

Total refers to the sum of all patients in each column or row; agreement refers to the total number of patients with concordant results. The denominator of the percentage calculation is the number of total patients for each group or subgroup.

# Table S5**.** Concordance of historical slides and re-stained slides scored at the local sites for HER2 IHC score and HER2 expression level.

| **HER2 IHC score** | **Historical slides rescored at local sites** | | | | | | | |  |
| --- | --- | --- | --- | --- | --- | --- | --- | --- | --- |
|  | **0** | **1+** | | **2+** | | **3+** | | **Total** | |
| **Re-stained slides scored at local sites** |  |  | |  | |  | |  | |
| 0 | 63 (87.5%) | 34 (42.5%) | | 2 (2.5%) | | 0 (0.0%) | | 99 (37.1%) | |
| 1+ | 7 (9.7%) | 43 (53.8%) | | 24 (29.6%) | | 0 (0.0%) | | 74 (27.7%) | |
| 2+ | 1 (1.4%) | 3 (3.8%) | | 53 (65.4%) | | 0 (0.0%) | | 57 (21.3%) | |
| 3+ | 1 (1.4%) | 0 (0.0%) | | 2 (2.5%) | | 34 (100.0%) | | 37 (13.9%) | |
| Missing | 1 | 0 | | 2 | | 0 | | 3 | |
| Total | 73 | 80 | | 83 | | 34 | | 270 | |
| Agreement | 63 | 43 | | 53 | | 34 | | 193 | |
| Kappa coefficient (95% CI) |  |  | |  | |  | | 0.62 (0.55–0.70) | |
| Quadratic weighted Kappa coefficient (95% CI) |  |  | |  | |  | | 0.84 (0.79–0.89) | |
|  |  |  | |  | |  | |  | |
| **HER2 expression level** | **Historical slides rescored at local sites** | | | | | |  | | |
|  | **HER2 IHC 0** | | **HER2-low** | | **HER2-positive** | | | **Total** | |
| **Re-stained slides scored at local sites** |  |  | |  | |  | |  | |
| HER2 IHC 0 | 63 (87.5%) | | 36 (24.8%) | | 0 (0.0%) | | | 99 (37.1%) | |
| HER2-low | 8 (11.1%) | | 108 (74.5%) | | 0 (0.0%) | | | 116 (43.4%) | |
| HER2-positive | 1 (1.4%) | | 1 (0.7%) | | 50 (100.0%) | | | 52 (19.5%) | |
| Missing | 1 | | 2 | | 0 | | | 3 | |
| Total | 73 | | 147 | | 50 | | | 270 | |
| Agreement | 63 | | 108 | | 50 | | | 221 | |
| Kappa coefficient (95% CI) |  | |  | |  | | | 0.73 (0.65–0.80) | |
| Quadratic weighted Kappa coefficient (95% CI) |  | |  | |  | | | 0.82 (0.76–0.87) | |

Note: the top half of the table computes the concordance of HER2 IHC score between re-stained slides and rescored historical slides, both of which are scored at the local sites; the bottom half of the table computes the concordance of HER2 expression level between re-stained slides and rescored historical slides, which are scored at the local sites.

Total refers to the sum of all patients in each column or row; agreement refers to the total number of patients with concordant results; Kappa coefficient refers to the overall agreement between re-stained slides and historical slides scored the local sites; quadratic weighted Kappa coefficient takes into consideration of the levels of disagreement between groups. The denominator of the percentage calculation is the number of total patients or subgroup.

# Table S6**.** Concordance between re-stained slides scored at the leading center and historical slides rescored at the local sites for HER2 IHC score and HER2 expression level.

| **HER2 IHC score** | | **Historical slides rescored at local sites** | | | | | | | | | | |  | |
| --- | --- | --- | --- | --- | --- | --- | --- | --- | --- | --- | --- | --- | --- | --- |
|  |  | **0** | | **1+** | | | **2+** | | | **3+** | | **Total** | | |
| **Re-stained slides scored at the leading center** | |  | |  | | |  | | |  | |  | | |
| 0 | | 65 (89.0%) | | 31 (38.8%) | | | 4 (4.9%) | | | 0 (0.0%) | | 100 (37.2%) | | |
| 1+ | | 7 (9.6%) | | 47 (58.8%) | | | 35 (42.7%) | | | 0 (0.0%) | | 89 (33.1%) | | |
| 2+ | | 0 (0.0%) | | 2 (2.5%) | | | 40 (48.8%) | | | 1 (2.9%) | | 43 (16.0%) | | |
| 3+ | | 1 (1.4%) | | 0 (0.0%) | | | 3 (3.7%) | | | 33 (97.1%) | | 37 (13.8%) | | |
| Missing | | 0 | | 0 | | | 1 | | | 0 | | 1 | | |
| Total | | 73 | | 80 | | | 83 | | | 34 | | 270 | | |
| Agreement | | 65 | | 47 | | | 40 | | | 33 | | 185 | | |
| Kappa coefficient (95% CI) | |  | |  | | |  | | |  | | 0.57 (0.50–0.65) | | |
| Quadratic weighted Kappa coefficient (95% CI) | |  | |  | | |  | | |  | | 0.82 (0.77–0.87) | | |
|  | |  | |  | | |  | | |  | |  | | |
| **HER2 expression level** | | **Historical slides rescored at local sites** | | | | | | | | |  | | | |
|  |  | **HER2 IHC 0** | | | **HER2-low** | | | **HER2-positive** | | | | **Total** | | |
| **Re-stained slides scored at the leading center** | |  | |  | | |  | | |  | |  | | |
| HER2 IHC 0 | | 65 (89.0%) | | | 35 (24.0%) | | | 0 (0.0%) | | | | 100 (37.2%) | | |
| HER2-low | | 7 (9.6%) | | | 110 (75.3%) | | | 0 (0.0%) | | | | 117 (43.5%) | | |
| HER2-positive | | 1 (1.4%) | | | 1 (0.7%) | | | 50 (100.0%) | | | | 52 (19.3%) | | |
| Missing | | 0 | | | 1 | | | 0 | | | | 1 | | |
| Total | | 73 | | | 147 | | | 50 | | | | 270 | | |
| Agreement | | 65 | | | 110 | | | 50 | | | | 225 | | |
| Kappa coefficient (95% CI) | |  | | |  | | |  | | | | 0.74 (0.67–0.81) | | |
| Quadratic weighted Kappa coefficient (95% CI) | |  | | |  | | |  | | | | 0.82 (0.77–0.88) | | |

Note: the top half of the table computes the concordance of HER2 IHC score between re-stained slides scored at the leading center and historical slides scored at the local sites; the bottom half of the table computes the concordance of HER2 expression level between re-stained slides scored at the leading center and historical slides scored at the local sites.

Total refers to the sum of all patients in each column or row; agreement refers to the total number of patients with concordant results; Kappa coefficient refers to the overall agreement between re-stained slides scored at the leading center and historical slides rescored at the local sites; quadratic weighted Kappa coefficient takes into consideration of the levels of disagreement between groups. The denominator of the percentage calculation is the number of total patients or subgroup.

# Table S7**.** Histopathological and clinicopathological characteristics in the rescored/FUSCC group.

|  | **HER2 IHC 0**  **(*n* = 682)** | **HER2-low**  **(*n* = 1,563)** | **HER2-positive**  **(*n* = 622)** | **Total**  **(*N* = 2,869)** |
| --- | --- | --- | --- | --- |
| Primary tumor, *n* (%) |  |  |  |  |
| T0 | 1 (0.1%) | 0 (0.0%) | 0 (0.0%) | 1 (0.0%) |
| T1 | 301 (44.1%) | 700 (44.8%) | 228 (36.7%) | 1,229 (42.8%) |
| T2 | 271 (39.7%) | 743 (47.5%) | 334 (53.7%) | 1,350 (47.1%) |
| T3 | 16 (2.3%) | 39 (2.5%) | 20 (3.2%) | 79 (2.8%) |
| T4 | 0 (0.0%) | 6 (0.4%) | 2 (0.3%) | 6 (0.2%) |
| Tis | 4 (0.6%) | 3 (0.2%) | 0 (0.0%) | 7 (0.2%) |
| Tx | 89 (13.0%) | 72 (4.6%) | 40 (6.4%) | 201 (7.0%) |
| Total | 682 | 1,563 | 622 | 2,869 |
| Regional lymph nodes, *n* (%) |  |  |  |  |
| N0 | 412 (60.4%) | 946 (60.5%) | 383 (61.6%) | 1,742 (60.7%) |
| N1 | 134 (19.6%) | 367 (23.5%) | 129 (20.7%) | 631 (22.0%) |
| N2 | 39 (5.7%) | 118 (7.5%) | 49 (7.9%) | 206 (7.2%) |
| N3 | 18 (2.6%) | 69 (4.4%) | 30 (4.8%) | 117 (4.1%) |
| NX | 79 (11.6%) | 63 (4.0%) | 31 (5.0%) | 173 (6.0%) |
| Total | 682 | 1563 | 622 | 2869 |
| Tumor size, *n* (%) |  |  |  |  |
| ≤2 cm | 362 (53.2%) | 748 (48.2%) | 242 (39.3%) | 1,352 (47.4%) |
| >2 cm | 318 (46.8%) | 804 (51.8%) | 374 (60.7%) | 1,498 (52.6%) |
| Missing | 2 | 11 | 6 | 19 |
| Total | 682 | 1,563 | 622 | 2,869 |
| Number of positive lymph nodes |  |  |  |  |
| N (Missing) | 681 (1) | 1,562 (1) | 621 (1) | 2,866 (3) |
| Mean (SD) | 1.2 (3.34) | 1.6 (4.04) | 1.8 (4.32) | 1.6 (3.95) |
| Median (P25, P75) | 0.0 (0.0, 1.0) | 0.0 (0.0, 1.0) | 0.0 (0.0, 1.0) | 0.0 (0.0, 1.0) |
| Min, Max | 0.0, 38.0 | 0.0, 50.0 | 0.0, 32.0 | 0.0, 50.0 |
| Histological types, *n* (%) |  |  |  |  |
| Invasive ductal | 629 (92.2%) | 1,426 (91.2%) | 583 (93.7%) | 2,640 (92.0%) |
| Other | 53 (7.8%) | 137 (8.8%) | 39 (6.3%) | 229 (8.0%) |
| Total | 682 | 1,563 | 622 | 2,869 |
| Histopathological grade, *n* (%) |  |  |  |  |
| Well differentiated (G1) | 48 (7.0%) | 153 (9.8%) | 3 (0.5%) | 204 (7.1%) |
| Mod. differentiated (G2) | 379 (55.6%) | 933 (59.7%) | 223 (35.9%) | 1,537 (53.6%) |
| Poorly differentiated (G3) | 211 (30.9%) | 407 (26.0%) | 373 (60.0%) | 991 (34.5%) |
| Not assessable (GX) | 44 (6.5%) | 70 (4.5%) | 23 (3.7%) | 137 (4.8%) |
| Total | 682 | 1563 | 622 | 2869 |

FUSCC, Fudan University Shanghai Cancer Center; G, grade; Mod. moderately.
